# Emerging Opportunities for Synthetic Biology in Agriculture

**DOI:** 10.3390/genes9070341

**Published:** 2018-07-06

**Authors:** Hugh Douglas Goold, Philip Wright, Deborah Hailstones

**Affiliations:** 1 Department of Molecular Sciences, Macquarie University, North Ryde, NSW 2109, Australia; 2 New South Wales Department of Primary Industries, Locked Bag 21, 161 Kite St, Orange, NSW 2800, Australia; philip.wright@dpi.nsw.gov.au; 3 New South Wales Department of Primary Industries, Elizabeth Macarthur Agricultural Institute, Woodbridge Road, Menangle, NSW 2568, Australia; deborah.hailstones@dpi.nsw.gov.au

**Keywords:** agriculture, microbial, plants, primary industries, synthetic biology

## Abstract

Rapid expansion in the emerging field of synthetic biology has to date mainly focused on the microbial sciences and human health. However, the zeitgeist is that synthetic biology will also shortly deliver major outcomes for agriculture. The primary industries of agriculture, fisheries and forestry, face significant and global challenges; addressing them will be assisted by the sector’s strong history of early adoption of transformative innovation, such as the genetic technologies that underlie synthetic biology. The implementation of synthetic biology within agriculture may, however, be hampered given the industry is dominated by higher plants and mammals, where large and often polyploid genomes and the lack of adequate tools challenge the ability to deliver outcomes in the short term. However, synthetic biology is a rapidly growing field, new techniques in genome design and synthesis, and more efficient molecular tools such as CRISPR/Cas9 may harbor opportunities more broadly than the development of new cultivars and breeds. In particular, the ability to use synthetic biology to engineer biosensors, synthetic speciation, microbial metabolic engineering, mammalian multiplexed CRISPR, novel anti microbials, and projects such as Yeast 2.0 all have significant potential to deliver transformative changes to agriculture in the short, medium and longer term. Specifically, synthetic biology promises to deliver benefits that increase productivity and sustainability across primary industries, underpinning the industry’s prosperity in the face of global challenges.

## 1. Synthetic Biology and the Primary Industries, Early Adoption of Disruptive Technology

Synthetic biology was propelled into prominence in the late 2000s when the costs of DNA sequencing and DNA synthesis became both cheap and fast enough to facilitate a paradigm shift in the way molecular sciences are conducted. Rather than taking a single gene approach (deletions or insertions), engineering principles were applied to biology and complex multigene constructs such as pathways and whole genomes could be generated. Identification of the genes essential for a minimal bacterial genome [1] and related projects such as genome transplantation [2], facilitated complex projects synthesizing whole bacterial genomes [3] and transplanting them back into bacterial cells [4]. These projects and other developments such as CRISPR/Cas9-mediated gene editing have enabled myriad, previously impossible tasks to become not only feasible but relatively simple [5,6]. Whole eukaryotic genomes are now being designed, the most ambitious effort to date being the design and construction of a synthetic yeast (*Saccharomyces cerevisiae*) genome, the Yeast 2.0 project [7,8,9,10,11,12,13]. However, synthetic genomics is just one example of synthetic biology. Metabolic engineering has been galvanized by the advances in genome technologies, with industrial scale production of complex metabolites from heterologous and at times de novo pathways becoming common [14,15,16]. New biosensors (genetically-encoded sensors for biological or non-biological stimuli) and increasingly complex genetically encoded circuits are being realized for a growing number of applications [17,18,19,20], there are many other emerging fields. The synergistic effect of these developments has positioned synthetic biology as a disruptive technology about to deliver significant outcomes to global agriculture. 

Primary industries such as agriculture, fisheries and forestry, have historically benefited directly from advances in genetic research. About half of the 1–3% annual increase in productivity in crops [21] and livestock [22] to date is estimated to have been driven by enhanced genetics, with rates of genetic gain predicted to more than double with the implementation of emerging molecular technologies. The sector has a strong history of rapid uptake of transformative innovation, for example worldwide between 1996 and 2013 more than 110 and 195 million tons of additional soybean and maize production, respectively, was attributed to positive yield effects of genetic technologies [23]. As an early adopter, the global agriculture industry is expected to be one of the major beneficiaries of synthetic biology [21]. The sector also faces significant challenges including increasing global population and technological innovations are key to meeting concomitant increases in demand for food and agricultural products [21,24]. Further challenges to agriculture include changing diets and more discriminating customers in a wealthier world, industry adaptation to digital and genetic technology, carbon constraints, environmental and animal welfare legislation, the increasing focus on ‘food as medicine’ and its ethical production, and changing risk profiles associated with globalization and climate variability [22,25]. Synthetic biology can provide tools to address many of these challenges and as an early adopter, the industry is likely to be a major beneficiary of the fast evolving global bioeconomy [26]. While synthetic biology is a broad domain and comprises many new, emerging and at times disparate fields, we aim to provide an overview of some key aspects of synthetic biology and how they may impact on primary industries, particularly agriculture.

## 2. Biosynthesis of High Value Plant Metabolites in Microorganisms

Yeast has served as a classic platform for metabolic engineering, due to the ease with which it can be modified, its rapid growth rates, the prevalence of infrastructure and industries relating to yeast and fermentation, its potential for high productivity, and its capacity to handle large genetic constructs [27,28,29]. Similarly, the simplicity and productivity of bacteria mean they are also regularly used as production hosts for medical and non-medical bio-products [15,30]. Plants are the source of a plethora of high value compounds, such as medicines [31], flavourings [32], and oils [33]; and so forth. However, their utility as metabolite producers brings caveats such as their dependence on arable land and water, long generation times, and seasonality. As aquatic photoautotrophs, algae are a natural alternative to plants with faster generation times and independence from arable land; however, the costs of cultivation and upscale of algae are prohibitively high [34,35]. Finally, challenges pertaining to directly engineering plants lie with long generation times [36], scalability, and large polyploid genomes (such as hexaploid wheat [37]). Transplantation of multigene pathways to foreign microbes, such as yeast, offers a useful trade-off, where plant derived carbon feedstocks can be converted with high efficiency to specific plant metabolites, and biochemical pathways can be rapidly augmented and optimized [38]. 

There are many examples of plant compounds being commercially synthesized in micro-organisms in this manner. The production in yeast of semi-synthetic artemisinin, a potent anti-malarial compound originally sourced from the plant *Artemisia annua*, was achieved by gradually addressing cytotoxicity and bottlenecks in the biosynthetic pathway [31]. Similarly, the production of the fragrant raspberry ketone was achieved by combining optimal enzymes for the pathway from a broad range of plant genomes to give a strain of yeast that produced a titer of over 7.5 mg/L [39], compared to 1–4 mg produced per kg of raspberries. Cannabinoids, opioids and cocoa butter are further examples of complex commodities produced in yeast from pathways that have been successfully transferred to yeast to satisfy pre-existing markets [40,41,42,43,44].

The cost of developing newly engineered microbes has reached an all-time low due to the combined decrease in the cost of DNA synthesis, DNA sequencing, and the increased throughput afforded by automation of the process of strain development [45]. Genome foundries, such as those at Amyris and Gingko Bioworks, attest to the feasibility and profitability of using microbial hosts to produce specific plant commodities resulting in market stability for at times unstable seasonal plant commodities [46]. Expressing plant pathways in non-plant hosts might affect land-use and provide new opportunities for domestic production [47]. Synthesis of plant compounds in microbial hosts releases agricultural production from the traditional paradigms of seasonality, resulting in plant derived commodities being available year-round and derived from a wide range of carbon sources (e.g., agricultural wastes) and potentially avoiding other constraints, such as frosts, plant pathogens and food safety. 

The relatively low cost and ease with which microbes can be developed to generate commodities that are generally produced by plants may change the way plantation decision-making is made on a global scale. As this technology becomes increasingly accessible, it is possible that practices could shift from large-scale cultivation of crops used for a single fragrance or flavouring, for example, to the cultivation of crops such as sugar cane, or any other crop that can be efficiently processed into a feedstock to supply carbon to microbial fermentations. These carbon feedstocks could then be used to generate a wider variety of synthetic agricultural commodities using bioprocesses derived from microbial systems, dependent on market conditions at any particular time. This could also potentially impact on import/export markets by facilitating domestic production of commodities unsuitable for domestic production in plants, or off-season production. These innovations could underpin economic growth and deliver new water use efficiencies through for example productive recycling of nutrient rich waste water, improving industry resilience and productivity in agriculture. 

## 3. Biosensors and Molecular Circuitry: A Reductionist View of Biology

One interpretation of synthetic biology could be that it is a reductionist view of biology through the lens of an engineer. By looking at a cell as a collection of inputs, processes and outputs it is possible to view the cell as one views circuit diagrams. For instance, a plant leaf cell can be reduced to the input of light, and output of carbohydrates. The genetics of this system are broken down into a gene that confers perception of light, and a metabolic pathway for carbohydrates. When the light genes are activated, the cell activates the carbohydrate synthesis pathway, feeding the rest of the plant. This concept can be extrapolated to a myriad of potentially exploitable systems in biology. 

**Figure 1:**
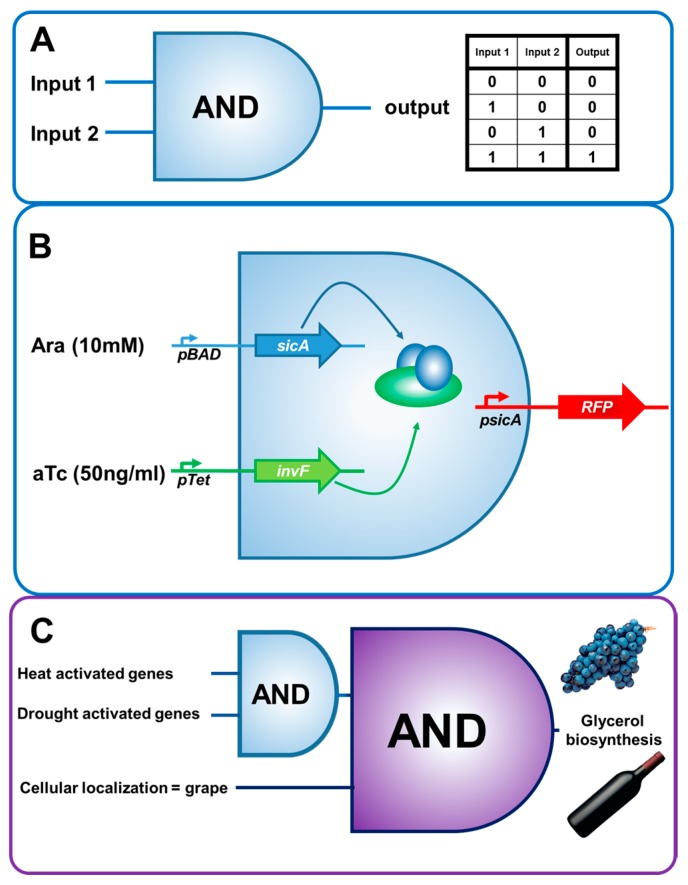
(**A**) AND gate, two inputs, if either or both input is present, the output is present, and the corresponding truth table applies. (**B**) Functional AND gate in *Salmonella typhimurium*, *pBAD* is activated by arabinose (Ara), *pTet* is activated by anhydrotetracycline (aTc). SicA is a chaperone (blue), and InvF is a transcription factor (green). If both inducing agents are present, sicA and invF will be produced in the cell, activate the *psicA* and RFP (red fluorescent protein) will be expressed. (**C**) Heat and drought activated genes could act as an AND gate input for a second AND gate. When all three conditions are satisfied glycerol biosynthesis could occur in the grape changing the grape glucose content, and thus wine ethanol concentration. Figure adapted from Voigt et al. [18].

The first element of a genetic circuit, biosensors (genetically-encoded sensors), are a potentially transformative field of synthetic biology with promise for agriculture in their own right. A biosensor refers to a genetically encoded element, such as a promoter or a protein which can react to exogenous stimuli, and create an output, classically gene expression [48]. The range of stimulatory molecules and conditions available to biosensors is extensive, with microbial biosensors reported for organic acids [48], carbohydrates [49], coenzyme B12 [50], heavy metals [51], amino acids [52], light [53], pathogens [54] and plant hormones [55] amongst other simple molecules and inputs. Downstream, signalling from a biosensor can be linked to a host of genetically-encoded elements, such as novel receptors, deactivated Cas9 and derivatives (capable of binding but not cleaving DNA) [17] and transcription factors [18] to elicit outcomes. The simplest circuitry comprises the AND gate, where *condition 1* and *condition 2* must be active for *outcome 1* to be activated (Figure 1A). Conditional stimuli (such as chemical stimuli, light, drought and or temperature) are perceived by biosensors, such as receptors and processed by elements such as (among others) chaperone molecules, and transcription factors (Figure 1B). Using the example of the vine grape, *Vitis vinifera*, ethanol content in wine is often higher in wine harvested from warm dry climates. High-ethanol wine is often undesirable, as ethanol can have negative impacts on wine flavour [56], increase ill effects in consumers and escalate product pricing where beverage taxes are indexed to alcohol content [57]. This could be mitigated by introduction of layered AND logic circuits, which would be active only in the grape, and during warm and dry periods to produce glycerol which is not fermented into ethanol and does not have a large impact on sensory perception of wine (Figure 1C). The term ‘Smart plant’ has been used to describe plants that have molecular circuitry, which will enable them to adjust appropriately to their environment [20].

Inverters, XORs (exclusive OR gates) transducers, oscillators, and many more logic operations can be translated into biological systems all with different potential outcomes; together these create a highly powerful toolbox for biological design [17,20,58]. The application of molecular circuitry depends on the combination of sensory inputs and outputs. For example, circuits have been used to identify subpopulations of engineered microbial cell factories [48,59], and to rapidly identify pollutants and toxins [51] and microbial pathogens in a clinical setting [54]. 

Returning to the previous example of *S. cerevisiae* engineered with the raspberry ketone pathway, where mutagenesis or breeding strategies involving the parental producer can create a varied population of raspberry ketone producer strains. A biosensor could be used to help identify the most productive strains in a population, as has been demonstrated in yeast cells with a ratiometric biosensor for para-hydroxybenzoic acid (PHBA) inside the cell [46]. In this case, the biosensor was used in conjunction with a rational strain engineering approach to isolate PHBA-producing strains of *S. cerevisiae* accurately when they represented only 0.01% of a population. These principles could be easily applied to the raspberry ketone yeast if an appropriate biosensor were to be developed [48]. 

An example of a biosensor with potentially broad reaching applications for the detection of a wide range of organic molecules ranging from food spoilage through to explosives is the modular G-protein coupled receptor (GPCR) system developed using chimeric BRET-biosensors (Bioluminescence Resonance Energy Transfer). The GPCR-BRET system was used to detect femtomolar levels of diacetyl using an odorant receptor protein from *Caenorhabditis elegans* [60]. The key potential of this system is its modularity, as many organisms across the domains of life have odorant and taste receptors which are GPCRs [61,62] and which could be exploited in this chimeric system.

Perhaps the biosensor most relevant to agriculture is the concept of the plant sentinel biosensor, an entire plant modified to detect and signal the presence of specific component in its immediate environment. By encoding a synthetic signal transduction pathway with a modular receptor, for example, plants can be programmed to respond to a wide variety of environmental pollutants, nutrients, abiotic stresses and other environmental factors [33]. A whole plant biosensor for the explosive 2,4,6-trinitrotolune (TNT) has been developed by engineering a bacterial receptor for TNT, a transmembrane kinase, and a response regulator to rapidly activate a de-greening gene circuit. The de-greening circuit inhibits new chlorophyll synthesis whilst simultaneously upregulating chlorophyll degradation genes, such that the sentinel plant rapidly changes when exposed to the cognate ligand (TNT) [33,63]. Other sentinel plants have been developed that signal exposure to gamma radiation [64] and to heavy metals [65]. *Arabidopsis* has been modified to signal when starved of phosphorous and similar modification of field crops could see them accurately and visually directing the temporal and spatial applications of fertilizers, improving application practices, reducing waste and improving sustainability [66]. This technology could be extended more broadly, with the development of sentinel plants that detect specific plant pests or pathogens, or abiotic stresses such as heat and water. Their simplicity would make for rapid incorporation into pre-existing agricultural farming systems, improving on-site decision making, delivering savings through reducing the use of agricultural chemicals and improving the sustainability of agricultural practises.

The imminent utility of molecular circuitry beyond microbes to plants and mammalian cells is demonstrated by the growth of these approaches to genetic design in these more complex systems [19,33,67,68], and offers significantly more potential than just the development of new crop varietals. Cells which are themselves ‘whole cell biosensors’ could transform primary industries [54,69]. Microbial biosensors can be expected to rapidly disrupt agricultural processes because they can be produced and deployed in a relatively short term [36], whereas sentinel biosensor plants could be used to signal a range of information as the foundation of intelligent cropping systems [66,70], transforming traditional agricultural practices. 

## 4. Opportunities for Plant-Based Agriculture through Innovations Drawn from Synthetic Biology

The major challenge to the implementation of synthetic biology in agriculture is the time and expense involved in propagation, transformation, and screening of higher plants. While there has been a boost to plant biotechnology following new developments such as CRISPR/Cas9-mediated gene editing [71], speed breeding [72], the sequencing of key genomes [37,73], and the growth of synthetic biology as a field [74], challenges remain. For instance, the immense size of plant genomes and their polyploidy (wheat, for example, has an hexaploid > 15 Gb genome [37]) have to now limited the effectiveness of site specific genetic manipulation. Also, plants generally have poor homology directed recombination (HDR) mechanisms compared to microbes [75]. It is also important for primary industries to remain aware of consumer attitudes towards genetic manipulation particularly in foods. 

A long standing target for improvement in plant based agriculture is nitrogen fixation [76,77]. Nitrogen in a bioavailable form for crops is incredibly expensive to produce and environmentally costly, using 1% of the total annual world energy expenditure [78]. While plants are unable to fix atmospheric nitrogen, microbes can and do, particularly rhizobia in legumes. A joint venture by Gingko Bioworks and Bayer, Joyn Bio, is targeting the reduction of global fertilizer use by one third [79]. By engineering the plant microbiome, they are seeking to improve nitrogen fixation in crop associated microbial species. Similarly, considerable efforts are underway to introduce direct nitrogen fixation into higher plants [80] and to introduce novel symbiotic associations with nitrogen-fixing bacteria [77]. 

Despite major challenges facing plant synthetic biology, the potential in a plant setting has been demonstrated. Photosynthesis drives agriculture and is the sole defining and unifying feature of green lineage organisms. It is, however, inherently inefficient, with a theoretical maximum efficiency of ~11% but typically not exceeding a few percent [81], thus providing many potential targets for synthetic biology to improve outcomes. For instance, the introduction of cyanobacterial carboxysomes into the chloroplast, could potentially overcome the inherent suboptimal activity of RuBisCO, the CO_2_-fixing enzyme in photosynthesis [82]. The feasibility of this approach has been successfully demonstrated by the localization of β-carboxysomal proteins that self-assemble into empty carboxysomal microcompartments in *Nicotiana benthamiana* chloroplasts [83]. This potential to increase the capacity and efficiency of plants to fix atmospheric carbon has clear implications for agricultural productivity and natural resource management.

Other opportunities include the potential to improve the nutritional value of foods, for example, through the development of carotenoid-enriched functional crops and oilseed crops with boosted levels of omega 3 fatty acids. Metabolic rewiring could be used to greatly increase the accumulation of carotenoids with nutritional and health-promoting activity, as recently demonstrated in proof of concept experiments [84]. Altering the protein quality control systems of plastids, which regulate protein turnover, has been shown to modify the carotenoid profile of tomato fruits, suggesting that this pathway could be manipulated to breed fruit crops with designed carotenoid content [85]. Furthermore, the synthetic control of plastid identity (i.e., the ability to convert one plastid type into another) is an ambitious, not yet demonstrated, approach that has been proposed to develop new carotenoid-enriched crops [86]. Similarly, metabolic engineering of traditional seed crops such as canola with genes to improve the nutritional quality of fatty acids can improve the nutritional qualities of harvested oils. This has been achieved in *Arabidopsis thaliana*, using seven enzymes from five different organisms, the yeasts *Lanchancea kluyveri*, and *Pichia pastoris*, and the algae *Micromonas pusilla* and *Pyramimonas cordata*, and *Pavlova salina* [87]. This work was then repeated in *Camelina sativa*, and in both cases demonstrates a significant increase in the nutritional profiles of oils in terrestrial higher plants [88]. 

Another opportunity for plant-based agriculture lies with land use. Globally land use is limited by availability, suitability for exploitation by commercial agriculture, and contamination by industrial processes rendering potentially arable land contaminated. Two potential strategies to address this issue are bioremediation using microbes, and engineering plants to grow in non-arable land. The first, bioremediation, is the use of biological systems to change an environment. Wild isolates of organohalide respiring bacteria (ORB) such as *Desulfmonile tiedjei* have been used for the bioremediation of organohalides with some success [89]. Extension of this concept to rational engineering approaches pertinent to agriculture are also being undertaken by researchers to convert microbes such as *S. cerevisiae* and *Escherichia coli* into potential bioremediation agents [90,91]. These are capable of bioremediation of heavy metal contamination, degradation of toxic aromatic compounds, and biomass based sugars [90]. The second strategy, engineering new cultivars suitable for non-conventional environments is increasingly possible due to newly available genome sequences of different cultivars, and novel or obscure organisms Using the sequences of hardy cultivars, plants can be reverse engineered to tolerate abiotic stresses [92,93]. Reverse engineering of traits such as halotolerance from candidate plants like the aquatic and halophilic angiosperm *Zostea marina* into crop plants will potentially play a transformative role in biological remediation areas of the world affected by salinity, and potentially will aid in the regeneration of non-arable land [94]. Similar reverse engineering strategies could be used to employ proven microbial solutions to particular problems directly into higher plants [90,95].

A comprehensive strategy to harness the potential of synthetic biology will deliver the next-generation of improved agricultural crops. The ability to selectively enhance the productivity and nutritional value of crop plants will address impending global challenges such as increased demand for food and fiber due to population growth, the potential effects of climate variability, and emerging expectations amongst consumers about the health, provenance, environmental sustainability and ethical production of food.

## 5. Gene Drives: A Powerful Technology Accelerated by Gene Editing

The application CRISPR/Cas9 is ubiquitous across synthetic biology. As a useful tool, the technology has a range of implications for management of pests, pathogens and invasive species. Veterinary antibiotics are used to cure bacterial infections in animals and, more controversially, as growth promoters and prophylactics; the development of resistance to antimicrobial medicines is a significant issue for livestock industries globally [96]. CRISPR/Cas9 sequence specific antimicrobials have been demonstrated to specifically clear mice of antibiotic resistant *Staphylococcus aureus*, via the activity of Cas9 in the bacterial genome targeting the antibiotic resistance gene [97]. A natural extension of this technology, when commercially available, would be for CRISPR/Cas9 technologies to deliver novel antimicrobials for disease control in livestock. Cas9 mediated gene editing also has many implications for the poultry industry specifically. Editing the sex determination genes could simply and realistically eradicate the need for male culling, delivering significant benefits from an animal welfare perspective [98,99]. A food safety issue with poultry derivatives is the presence of the major allergens ovomucoid, ovalbumin, ovotransferrin and lysozyme. Genes for these proteins could be removed from the chicken genome to manage those allergy issues with no known impact on avian fertility and little impact on food quality [98]. Indeed ovomucoid homozygous mutants have been achieved using a mix of conventional breeding of heterozygous gene edited chickens [100].

**Figure 2:**
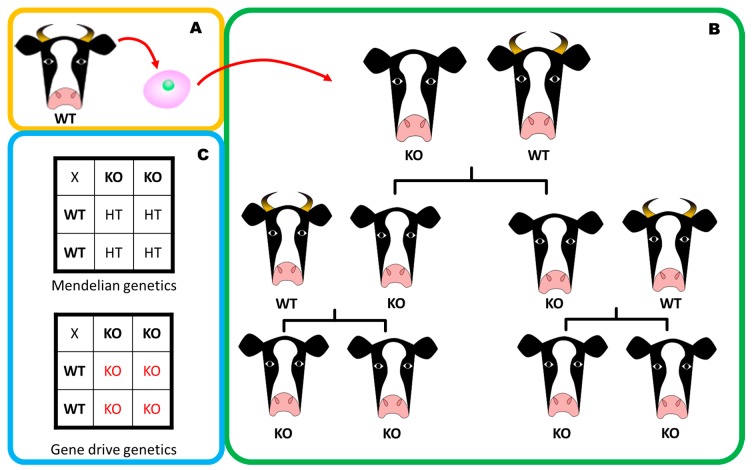
(**A**) The genome of an ideal milking Holstein is modified with a gene drive targeting the *polled* gene (responsible for horns in cattle) resulting in a *polled* gene-drive knockout cell line. (**B**) Somatic cell transfer produces knockout progeny which are raised and bred with the best milkers (wt for the *polled* gene), the gene drive ensures the breeding program yields exclusively knockout/hornless progeny. (**C**) Punnett squares demonstrate non-Mendelian genetics of gene drives and all heterozygous offspring in a conventional breeding setting become homozygous knockouts at the *polled* locus. Figure adapted from Gonen et al. [105].

Another key application of CRISPR/Cas9 to agriculture is the potential for nuclease-based gene drives to eradicate pest species [101], an area currently driven primarily by human health considerations in seeking to manage and eliminate the mosquitos that vector malaria using anti-insect gene drives. A classic example of a nuclease gene drive (Figure 2) is where a locus of interest is knocked out through replacement directed by CRISPR/Cas9. The gene drive will target the in-tact allele creating a double strand break. The DNA repair mechanism will use the knockout (gene drive) allele as a repair template, and replace the wildtype allele with a second copy of the knockout, resulting in a homozygous knockout [102]. The repair of the double-strand break will be conducted from the copy of the chromosomal DNA where the CRISPR/Cas9 construct is located, thus generating a homozygous knockout. This ensures that when the organism breeds, it will transfer one copy of the CRISPR/Cas9 construct to its progeny and despite them becoming heterozygotes by classical Mendelian genetics, the gene drive will self-replicate to match the wild-type chromosome, and create a homozygous knockout. Use of gene drives has been discussed as a potential means of eradicating wild populations of problem species including the house mouse, the European red fox, the feral cat, European rabbit, cane toad, black rat, and the European starling [103]. It is currently debated whether these could be used to remove insect pests from ecosystems entirely, but there are also other applications [101]. 

Gene drives could also be exploited for other applications. For example, the simulation of nine different breeding and editing scenarios demonstrated that genome editing in livestock breeding could deliver short, medium and long-term increases in genetic gain. The development of polled (or hornless) cattle could significantly improve both workplace safety and animal welfare in the farm environment, but the polled breeds currently available do not satisfy requirements for both meat and dairy products. Transcription activator-like effector nucleases (TALEN), a similar approach to CRISPR/Cas9, have been employed to knock-out the locus responsible for horns in cattle (Figure 2a), and somatic cell nuclear transfer then used to generate four lines of newly-hornless cattle (Figure 2b). Classical breeding to achieve this goal would take more than twenty years in dairy cattle, meaning that gene drive approaches could cost-effectively accelerate the breeding of new lines (Figure 2), delivering considerable savings and enhancing farm safety and animal welfare [104]. 

More broadly, gene editing and gene drives have been suggested as a potential mechanism for reverting atmospheric levels of CO_2_ and greenhouse gasses for example, by the use of non-photosynthetic CO_2_ capture pathways. This would include engineering pathways such as the reverse tricarboxylic-acid cycle, the Woods-Ljungdahl cycle, the hydroxybutyrate cycle, or new-to-nature pathways into microbes or plants. These pathways are capable of fixing atmospheric carbon, but not through the conventional means used by C_3_ or C_4_ plants [106]. Introduction of these biochemical pathways using CRISPR/Cas9 gene drives into wild populations or ecosystems could mitigate accumulation of greenhouse gasses such as CO_2_. Similarly, gene drives and gene editing could potentially address increasing humidity of arid ecosystems, mitigating the build-up of plastic waste, and the removal of pharmaceuticals and endocrine disruptors from trophic chains. These suggestions are on the basis of complex metabolic engineering approaches being propagated through wild microbiome populations using gene drives in engineered phages, or through horizontal gene transfer to effect an ecosystem-wide modification [107]. 

## 6. Whole Genome Approaches to Synthetic Biology: Synthetic Genomics

A natural progression that epitomizes the ideals of the synthetic biology revolution is the design and synthesis of whole genomes, which allows the engineering of new genomes to endow organisms with novel functions. 

Genome minimization is a hot topic [1,108] due to the possibility of generating more scientifically tractable simple model organisms. Many genomes are large and redundant and to date are poorly understood. Removal of large segments of these genomes does not necessarily render them inviable. For example the green alga *Chlamydomonas reinhardtii*, has a 120 Mb genome but can still function with deletions of up to 250 kb in a haploid cell [109]. Identification of the essential elements required for cellular function could reduce the metabolic burden of unnecessary or secondary genetic pathways, redirecting carbon to those delivering the best outcomes for an organism, such as those conferring improved nutrition (in a crop) or production of a particular high value metabolite (in an engineered microbe) [36]. The minimization of genomes to essential metabolism also provides a potential strategy to identify functions of gene products fundamental to different types of plant life, revealing likely targets for novel herbicides.

In the mammalian context, CRISPR/Cas9 has been used in a whole-genome approach by a process called multiplexing, whereby one targeting sequence corresponds to multiple sites across a genome. All porcine endogenous retroviruses (PERV) were removed from the pig genome using multiplexed CRISPR/Cas9 [102]. The resulting PERV-free fibroblasts were used in somatic cell nuclear transfer, generating PERV inactivated whole pigs. While the overall goal of this work was for to ensure healthy pig tissues for xenotransplantation, there are also some broad reaching implications of this work relevant to food safety and livestock health [110]. 

The Yeast 2.0 project is a contemporary and highly collaborative example of a whole-genome approach to synthetic biology. A genome design is synthesized and then built into a wild-type cell, 30–60 kb at a time [7,8,9,10,11,111,112]. One of the many design elements included in the Yeast 2.0 genome, the ‘synthetic chromosome rearrangement and modification by *LoxP*-mediated evolution’ (SCRaMbLE) system offers a particularly useful feature [12,112] where Lox-P-sym sites, which are symmetrical Cre-recombinase sites, are inserted between all non-essential genes. This allows for genome wide rearrangements (inversions, translocations, deletions, and duplications) and gives the cell a new capacity as a chassis for genome engineering [113,114]. This system has been shown to have produced numerous β-carotene and violacein producing mutants in a process called SCRaMbLE-in [113]. Combination of a biosensor, with SCRaMbLE, and engineered metabolic pathways including Lox-P-sym sites would allow for strains which are able to generate very high quantities of particular metabolites, and or grow in adverse conditions. Through biosensors and metabolic engineering, SCRaMbLE holds promise for creating a new synthetic microbe which could have widespread applications ranging from cheap commodity synthesis to improved bioremediation [51,90,113,114].

Another genome-scale technology, genetic ‘recoding’, removes all instances of a particular codon for an amino acid from a genome. The codon can then be used in the new organism to specify different, or novel, and unnatural amino acids [115,116]. The concept has been demonstrated to be robust, with over 1557 leucine codons replaced across 176 genes in a *Salmonella typhimurium* strain, with no impact on cell growth [117]. Furthermore, recoding has been demonstrated as a potential mechanism to confer viral pathogen resistance by blocking effective horizontal gene transfer [118] however the more significant implication of this technology is the control of the flow of genetic information. Genetically encoded ‘speciation’ in yeast [119], is a part of a larger push towards improving biocontainment using synthetic biology, with broad reaching implications on mitigating potential unforeseen consequences of release of synthetic organisms [120]. 

Whole genome-based strategies alone, or in combination with other key technologies can effect great outcomes ranging from goals of biocontainment, to completely novel genomes with new functions not present in their parental templates. While the majority of approaches in this space focus on microbial species, the growth of new methodologies and capacities in synthetic biology is bringing the field closer to crops and higher plants. These form a suite of developing disruptive technologies with the potential to deliver a range of positive impacts including integrated pest and disease management, and delivering an accelerated understanding of gene function. 

Overall, whole genome approaches to synthetic biology appear to hold great potential for agriculture. As CRISPR/Cas9-mediated genome editing moves from single insertions/deletions to whole genome modifications, even more complex synthetic genomics projects are on the horizon. Whole genome design and synthesis will be further accelerated through the Genome Write Project [121], delivering more elaborate, useful, and exciting projects to agriculture.

## 7. Regulation and Commercialization, the Next Challenges to Synthetic Biology

The promise associated with synthetic biology does come with risks that must be identified, mitigated and managed. Regulatory frameworks need to manage risks and ensure an appropriate balance between the industry’s enthusiasm to access new technologies and any concerns within the community more broadly. There is a lack of consistency in approach to regulation of GMOs (genetically modified organisms) internationally, Frameworks differ significantly between countries, and most have failed to keep pace with the rapid development of new genetic technologies. Many touch on topics related to synthetic biology [122] but few nominate synthetic biology directly. Synthetic biology and gene editing are consistently amongst the technologies most frequently drawn to the attention of the regulators.

Gene drives hold significant and immediate promise for integrated pest management, given their significant potential to spread traits across a breeding population. However, potential issues such as the incorrect identification of favorable alleles, the spread of gene drives from farmed populations to natural populations and the mutation of gene drive elements all pose unique concerns [105] and potentially require more significant risk assessment prior to, and containment and record keeping during implementation [123]. Currently gene drives would be classified as veterinary medicines or toxins according to US policies and regulations. However there are gaps in how these policies apply to synthetic biology (especially gene drives) [123]. Regulation must also seek to manage the risk of “dual use”, where a product developed for altruistic goals could be exploited for alternative and harmful outcomes [122]. Bench scientists are increasingly expected to have knowledge of these issues [13,32], although the rise of DIYbio (do-it-yourself biology), citizen scientists, and biohackers contribute to a steady growth in this area and create an increasing difficult regulatory environment [124]. 

Existing legislation will need to be updated to ensure that new capacities that can be delivered by synthetic biology and other transformative genetic technologies are accessed safely, as and when appropriate. To this end, Australia is currently undertaking its third review of its National Gene Technology Regulatory Scheme, established in 2000, following an earlier technical review. 

Beyond regulation, a secondary constraint to all transformative technologies is the reality that early innovations are not necessarily published in the public domain. This is not unique to synthetic biology, and reflects that innovations relating to potentially profitable endeavors are likely to be patented then commercialized. This impacts on ‘freedom to operate’ and may help explain the paucity of manuscripts in the international literature that deal with synthetic biology in agriculture. We note that the sales of fertilizers and agricultural chemicals are worth $160 billion and $40 billion a year respectively [79,125] and the market value of companies in the field such as Indigo Ag, demarcating research around water and nitrogen use efficiency, and Gingko Bioworks working on microbial fragrances, nutraceuticals, flavourings, and agriculture, and eligo biosciences which works working in the field of microbiome engineering [79,126]. 

The intellectual property footprint of these well-funded companies can pose barriers to dissemination of synthetic biology in the scientific community, whilst regulation, and deregulation may stymie access to the technology in the agricultural sphere.

## 8. Conclusions

The agricultural sector is often cited as potentially one of the major beneficiaries of synthetic biology, but a range of challenges in applying the technologies to higher plants and livestock, and potentially commercial interests, means there is little work in the public domain. The sector has a long history of early adoption of transformative innovation, including genetic technologies to decrease the use of pesticides and enhance social license. It is conservatively estimated that rates of gains in productivity and profitability that are directly attributed to genetic improvements in agriculture will be more than doubled by the new emerging genetic technologies. The development of new tools and more efficient and effective synthetic biology approaches has unlocked the potential to deliver outcomes to primary industries that range from new crop choices, productivity, agronomic efficiency, pest management animal welfare, and the nutritive value and safety of foods. The development of transformative technologies such as speed breeding, gene editing and whole genome synthesis are increasingly underpinned by reducing costs of DNA synthesis and the emergence of companies providing a wider range of services to generate and screen new DNA and organisms, stimulating discovery and innovation that will directly benefit agriculture. 

Synthetic biology is a disruptive and highly beneficial technology that promises to deliver benefits that will allow primary industries to address major global challenges such as the increasing demand for food, fiber, diversified diets and ethically sourced products; the depletion of natural resources such as soil and water; and in the face of increased trade and environmental change.
